# Yet they failed to do so: recommendations based on the experiences of NAOMI research survivors and a call for action

**DOI:** 10.1186/1477-7517-10-6

**Published:** 2013-04-18

**Authors:** Susan Boyd, NAOMI Patients Association

**Affiliations:** 1 Studies in Policy & Practice, University of Victoria, PO Box 1700 STN CSC, Victoria, BC, V8W 2Y2, Canada

## Abstract

**Background:**

This article highlights the experiences of a unique group. In January 2011, Dave Murray organized a group of participants from the North American Opiate Medication Initiative (NAOMI) heroin-assisted treatment clinical trials from 2005 to 2008 in the Downtown Eastside of Vancouver (DTES), B.C., Canada. The NAOMI Patients Association (NPA) is an independent group that currently meets every Saturday in the DTES. Currently, all members of the NPA are former participants in the heroin stream of the clinical trial. The NPA offers support, education, and advocacy to its members.

**Methods:**

Drawing on brainstorming sessions and focus groups that were conducted in the summer of 2011, this paper highlights the experiences of NPA members in their own words.

**Results:**

The findings provide a lens to understand how becoming a research subject for the NAOMI trial impacted the lives of NPA members, both positive and negative. The NPA members discuss ethics, consent, recommendations for future HAT programs and studies, and ongoing advocacy.

## The NAOMI Patients Association (NPA): participant support and advocacy

In January 2011, Dave Murray organized a group of participants from the North American Opiate Medication Initiative (NAOMI) heroin-assisted treatment clinical trial from 2005 to 2008 in the Downtown Eastside of Vancouver (DTES), B.C., Canada. The NAOMI Patients Association (NPA) is an independent group that currently meets every Saturday at Vancouver Area Network of Drug Users (VANDU). The NPA is associated with the British Columbia Association of People On Methadone (BCAPOM). From its inception to early 2012, all members of the NPA were former participants in the heroin stream of the NAOMI clinical trial. Many former NAOMI participants had a difficult time following the cessation of the NAOMI study. Dave Murray recognized this and as a result initiated the formation of the NPA, in January of 2011. The NPA began to meet weekly at VANDU to offer support, education, and advocacy for its members. The NPA has reached out to all former NAOMI participants in the heroin stream of the clinical trial. Although attendance at weekly meetings varies, the highest attendance at a meeting was 44 members. On average, 15 members gather each week. Currently, a number of NPA members are also research subjects in another clinical trial in Vancouver: Study to Assess Longer-term Opiate Medication Effectiveness (SALOME). The NPA’s Mission Statement below exemplifies the group’s ethics and concerns:

## NPA mission statement

We are a unique group of former NAOMI research participants dedicated to:

● Support for each other

● Advocacy

● Educating peers and the public

● Personal and political empowerment

● Advising future studies (heroin and other drugs) and permanent programs

● Improvements in consent and ethics

● The right to a stable life and to improvement in quality of life

The goal of NPA is to have alternative and permanent public treatments and programs, including heroin-assisted treatment (HAT).^a^

## NAOMI Patients Association study

In May 2011 the NPA decided to undertake their own research about their experiences as NAOMI research participants. They were particularly interested in recording their experiences during and following NAOMI and making recommendations for other heroin and drug substitution research experiments and programs. They met with co-author, Susan Boyd, in the spring of 2011 and decided to work together to conduct qualitative research: focus groups, individual interviews, brainstorming sessions, and writing workshops with NPA members. NPA members also wished to write a report about their experiences. This paper draws on the brainstorming session and focus groups that were conducted from April to June in 2011 [1], and events following the research.

The NPA research project received ethics approval from the University of Victoria, B.C (Protocol Number 11-052). All NPA participants were granted confidentiality and anonymity and signed a consent form prior to participating in the focus groups. However, all NPA members insisted that their first name be included on their creative writing. At the brainstorming session 20 NPA members identified several topics they wanted to discuss in later focus groups. The first focus group took place in May 2011 and 10 NPA members (four women and six men) discussed the topics identified in the brainstorming session. The second focus group took place in June 2011 and nine NPA members participated (six men and three women).^b^ Each focus group was audiotaped and transcriptions were made. After careful reading and coding of the transcripts, five primary themes were identified:

● Beneficial outcomes of being a participant in NAOMI

● Problematic outcomes of being a participant in NAOMI

● Ethics and Consent

● Creative writing/Everyday life

● Recommendations for other research projects and programs

This paper highlights the experiences of the NPA members in their own words. The themes identified above, except for the creative writing, are expanded upon in these pages. The findings presented in this paper provide a lens to understand how people addicted to narcotics navigate their lives in and outside of the DTES and how becoming a research subject for the NAOMI project impacted their lives, both positive and negative. The first section of the paper introduces readers to the DTES and a number of ethical issues, including the impact of research conducted in the area. This section is followed by guidelines and principles created by VANDU for researchers working in the DTES and elsewhere. The next section provides a brief history of Canadian drug policy, heroin-assisted treatment and the circumstances that led to the NAOMI clinical trial. Following are quotations, through which the NPA provides insight into the lives of their members during the NAOMI clinical trial both within and outside of their role as drug users and research subjects. Finally, NPA members outline guidelines for future drug substitution studies and programs, and ongoing advocacy. This paper concludes with the hope that future research and heroin treatment programs will benefit from the experiences, reflection, and advocacy of the NPA.

## The downtown eastside, research, and ethics

NPA meetings take place at VANDU, on East Hastings Street in the DTES. The NAOMI clinic was also located in the DTES. Although it was not always so, today the Downtown Eastside of Vancouver is Canada’s poorest urban neighbourhood [2]. According to the city of Vancouver, the DTES includes Gastown, Victory Square, Strathcona, Chinatown, industrial lands, and the Oppenheimer and Thornton Park areas. Just over 18,000 people live there [3] pp. 6,3. It is a racially diverse population of Aboriginals, Asians, Latinos/as and Caucasians [3] p. 9. The DTES has a number of single-room occupancy (SRO) establishments and a visible street scene [3] p. 14. The street scene is directly related to cutbacks at the federal, provincial, and local levels, leading to poverty and a lack of social housing and private space. Gentrification of the area has also made it more difficult for long-time residents to find safe, permanent, and affordable housing. For women, especially First Nations women, the DTES is also the site of much violence, often linked to the sex trade, but more generally, to everyday life [3] p. 25, [4-6]. The negative outcome of drug prohibition and the criminalization of heroin, cocaine, and other drugs is played out on the streets daily. Prohibition fuels an illegal market and, unlike in more privileged neighbourhoods, drug use and selling is more visible on the street in the DTES instead of hidden behind closed doors. This situation makes people more vulnerable to unwanted police attention and prison time, and sometimes drug-related violence [3] p. 24.

It is well documented that drug prohibition, a reliance on the criminal law to eliminate illegal drug production, selling, and use, has worsened the health and well-being of drug users [7]. The results of prohibition include increased imprisonment and the undermining of health services, including prevention and treatment services that would more effectively counter HIV and Hepatitis C epidemics and drug overdose deaths [7]. Effective countermeasures are undermined. (For example, Insite, the safe injection site in Vancouver, was challenged by the federal government which actively sought to close it down and challenged its legitimacy in court despite scientific evidence demonstrating its effectiveness.) Prohibition also fuels social and legal discrimination and stigma, and the marginalization of people who consume illegal drugs. Globally, law enforcement and civil initiatives over the last 100 years have led to increased incarceration, prison building, and the infringement of human rights [7]. Recently in Canada, the Conservative federal government enacted mandatory minimum sentencing for some drug offences. In March 2012, Bill C-10, the Safe Streets and Communities Act, was enacted.

The individual and social costs of this Act could be immense: families may be torn apart when parents are sentenced to prison; children may be apprehended by the state; and the loss of income for families may leave many destitute. At the weekly meetings, the NPA see daily that it is the poor and marginalized who suffer the most under prohibition; they, not an imagined “drug king pin,” are arrested, convicted, and sent to prison. Yet, prohibition is not uncontested. In the 1990s activists in the DTES came together to challenge the status quo and they continue to do so today.

The DTES gained national and worldwide attention in 1997 when a public health emergency was declared in response to the growing rates of HIV, Hepatitis C, and overdose deaths in the area. Stemming from those events, community activists played a major role in a social movement for change in the DTES, demanding an end to drug prohibition, more social supports, and the establishment of more harm reduction services, such as a safe injection site [8].

VANDU also emerged in 1997, the first drug user union in Canada. VANDU has long advocated for their members and for change in the DTES and to Canada’s drug laws and policy [9]. Due to these efforts and those of other community activists, the DTES has witnessed some changes since 1997. VANDU secured a permanent site and offers support, education, and advocacy for group members. Needle exchange expanded, the Portland Hotel Society provided more housing for people with addictions and/or mental illness, the first safe injection site, Insite, opened its doors in 2003, and a heroin prescription trial, NAOMI, opened its doors in 2005. Yet, for many residents, especially people who consume illegal drugs such as heroin and crack, the social conditions of their lives barely changed. Lack of affordable and safe housing, poverty, racial profiling, criminalization, violence, drug prohibition, and discrimination continue to shape the lives of people living in the DTES [3].

At the same time that activists in the DTES have striven to improve the conditions of people’s lives in the area and to advocate for change, health and social science researchers began to conduct studies in this area and many of the residents became research subjects [6,10-16]. Many of these studies made clear empirically what the residents already knew: a myriad of health and social factors detrimentally shape the lives of people in the DTES.

NPA members wanted to conduct their own research, in part because they had participated as research subjects in the NAOMI trial. However, for many NPA members, the NAOMI trial was not the first study in which they had participated. In the DTES of Vancouver, one of the only ways to access services or to make ends meet is to become a research subject. Research honorariums, bus passes, stipends, and for a short period, access to unadulterated legal heroin, are now familiar exchanges in the DTES.

In 2005, the Canadian HIV/AIDS Legal Network published, “*Nothing About Us Without Us” – Greater, Meaningful Involvement of People Who Use Illegal Drugs: A Public Health, Ethical, and Human Rights Imperative*[17]. This booklet responded to the negative impact of programs and research studies on people who use illegal drugs, including the participants’ exclusion from the development of studies, programs, and services. This booklet includes a manifesto by people who use illegal drugs: the authors recommend greater involvement of people who use drugs in the programs and services that affect their lives, as well as in broader policy and advocacy work on HIV/AIDS and Hepatitis C. A recent 2011 report by the World Health Organization and UNAIDS, *Ethical engagement of people who inject drugs in HIV prevention trials*, echoes the recommendations by Canadian HIV/AIDs Legal Network. They also recommend that research populations “participate in a meaningful way in each stage of a trial, including the earliest stages during which a study is conceptualized and research protocols are developed” [18], p. 7. In addition they recommend that all research subjects continue receiving treatment at the end of a trial if the care and treatment are effective [18], p. 26.

The issues surrounding research, ethics, and exploitation have long interested drug user groups and activists because they have themselves become research subjects or witnessed others participating in trials and studies (further on we highlight Dan Small’s and Ernest Drucker’s concerns about these issues in relation to NAOMI). Dara Culhane, a researcher and long-time resident in the DTES, also critiques the ethics of some of the research conducted in the DTES. She is critical of “data-mining,” which she defines as “researchers using research subjects principally as means to researchers’ ends” [19], p. 260. She argues data-mining has become a “central dynamic in everyday encounters between research*ers* and research*ed*” in the DTES even though residents have strongly protested against these interventions [19], p. 260. In the section below we draw extensively from Dara Culhane’s recent 2011 article published in *Anthropologica*[19]*.*

Culhane notes that the DTES has become an “internationally renowned centre for medical and pharmaceutical research on HIV/AIDS and addiction” dating back to the public health crises in 1997 [19], p. 261. She explains that along with harm reduction services came clinical trials (including NAOMI) and studies to prove their effectiveness and thus, “a research industry expanded dramatically” in the DTES [19], p. 261. Yet the social conditions of people’s lives there have for the most part worsened.

Culhane questions the role of many marginalized people in the DTES “to serve as research subjects” in varied social science studies, clinical trials, and art projects. She argues that for marginalized people, “access to public support and private philanthropy increasingly demands performing” and telling one’s story in exchange for “food, housing, health care, attention, affection, compassion and belonging.” Researchers, of course, benefit from “grants, publications, tenure and promotion” [19], p. 262. Culhane writes that the stories told are true and the pain they reveal is real. Many DTES researchers try hard to be ethical; however, the conditions of poverty and exploitation in which studies take place challenge researchers to find ways to support research subjects' autonomy and self-determination within projects themselves and within society as a whole.

Because people living in the DTES need income and services, these needs shape how their stories are told to researchers. Responding to these events in the DTES, the NPA decided to conduct their own research, to tell their own stories, in their own words. They adopted the principles of collaborative research and “*Nothing About Us Without Us*” [17]. These research perspectives offered an approach to make visible the experiences of the NPA members. These perspectives also emphasize “research for social change” and policy relevance [20]. During the research process, the issues of participation, ethics, and consent were discussed by the NPA. In these pages the NPA asks their readers: how can there be consent to participate in studies, to tell one’s story, including participation in the NAOMI clinical trial, when the social conditions of people’s lives are so compromised? How can research be initiated and guided by the experiences and knowledge of those most affected by drug prohibition? The NPA members speak to these issues and others in the following pages. In the fall of 2011, VANDU, including members of the NPA, created further guidelines and principles for researchers working with VANDU and the NPA. They note that research is conducted against the backdrop of the war on drugs:

● The drug war didn’t start because of a lack of research or “bad” research and we don’t think it will end because of “good” research. The active struggle of people oppressed by drug war policies and fighting for their liberation will be the decisive factor in ending the drug war. Researchers can play a positive role when they act as supporters, allies and partners of this movement for liberation.

● Research is political. Research is shaped by funding, by the career aspirations of researchers, by the political tendencies of research institutions, by government funding and intervention, by peer pressure and by class, racial and gender biases.

● The relationship between the researcher and the researched is not in and of itself empowering or liberating. It only becomes so when organized movements of the oppressed group play an active role in shaping and carrying out the research.

● Researchers should leave the organizations of oppressed people that they work with stronger than when they came in; if they don’t, they are part of the problem and not part of the solution [21].

The NPA adopted the words below to further guide their own research. They are written by long-time DTES activist, historian, and poet Sandy Cameron, and are an excerpt from his poem, *Telling Stories*.

*Telling Stories*

We need to tell our own stories.

If we don't tell our stories,

people with power

will tell our stories for us [22].

It is from this place that the NPA began their own research, to tell their own story in their own words. This paper also provides a brief history of Canadian drug policy and heroin-assisted treatment in order to contextualize the NPA’s experience.

## Canadian drug policy and heroin-assisted treatment

All of the NPA members were research subjects in the NAOMI clinical trial in Vancouver, B.C. The following section outlines briefly the history of drug policy in Canada, drug maintenance therapy, and the circumstances that led up to the NAOMI trial, followed by a summary of the research findings of the NAOMI trial.

Prior to the criminalization of narcotics in Canada in the early 1900s, opiate (and opiate derivatives) use was acceptable in society to treat a wide range of illnesses [23-25].^c^ In the 1700s and 1800s, settlers to Canada brought opium remedies, patent medicines, and elixirs to treat illness. Most settlers could not afford the services of a doctor; nor were doctors available in rural areas, which made up most of Canada then. Although Aboriginal healers had their own array of remedies, the practices of colonization led white settlers to eventually reject Aboriginal medicines and to use drugs with which they were already familiar, including a wide array of oral patent medicines that were made available at stores and through mail order at Eaton’s and Sears, Roebuck and Company [26]. Doctors heralded these drugs (opium, cocaine, and marijuana) in liquid form for their healing properties to control coughing, address gastro complaints, and treat severe pain. Opiates were advertised to appeal to women as caregivers to their families. In Canada, Britain, and the U.S., it was quite common for households to contain patent medicines and remedies that included opiates. In fact, prior to the criminalization of opium and heroin, the typical persons using opium or its derivatives, such as Laudanum, were law-abiding middle and upper class white women [23,24,26,27]. However, what was once considered a personal matter shifted in the late 1800s and early 1900s. In 1803, morphine was isolated from opium. This was the first time in history that a chemical compound was extracted from a plant. This event led to other scientists and pharmacists experimenting with an array of plant compounds, and eventually to the creation of synthetic drugs and our modern day pharmaceutical and chemical industry [28]. Heroin is a derivative of morphine. It is more potent than morphine in that it produces the same effect but with smaller doses. Heroin was marketed by Bayer Pharmaceutical Products in 1898. Early on, it was popular as a cough suppressant; its popularity and medical applications were illustrated by the advertisements for its use that appeared in medical journals in the early 1900s [25].

For the purpose of this paper, we only wish to point out that opiates and opiate derivatives were in common use in Canada prior to Canada’s first narcotic legislation, the Opium Act of 1908 and the Opium and Narcotic Act of 1911. The Opium Act was not enacted because of evidence that opiates caused physiological harm; rather it was enacted to control Chinese Canadians in western Canada, and its original focus was on the sale and manufacture of smoking opium and not the array of liquid-based opiate drugs that white settlers consumed [29]. From their inception, Canada’s drug laws have been racist, class-based, and gendered in their formation and application. It was assumed that the Opium and Narcotic Act, for example, would not be applied to white middle-class citizens [29]. The Act was passed and subsequent amendments criminalized other drugs. As the schedule of prohibited drugs expanded, so did penalties and police/RCMP budgets over the years. The most prominent feature of Canada's drug policy over the last century has been a reliance on the criminal law, also called “prohibition” [30]. These laws were enforced by the RCMP and other police forces in Canada. Police and RCMP also became key initiators of harsh drug policies [31,32].

Following the criminalization of opium, heroin, and a number of other drugs in the early 1900s, a new group of citizens became criminals and they had few options for obtaining legal and illegal narcotics. Unlike doctors in the U.K., Canadian doctors did not retain the right to prescribe narcotics for maintenance purposes and no publicly funded drug maintenance programs were set up. In the 1920s, the police focused on arresting and deporting Chinese Canadian residents convicted of possessing opium in smoking form and closing down opium dens. Eventually police attention shifted to white narcotic users, especially those who were poor and working class. As the opium dens closed, narcotic users switched to other drugs such as heroin bought on the illegal market. Prison time was often their fate as Canada’s drug laws became more and more harsh. In the 1940s the RCMP coined the phrase “criminal addict” to describe people addicted to illegal drugs. They wanted to make clear that first and foremost, these people were criminals; the RCMP promoted the idea that addiction was secondary and stemmed from having a “criminal lifestyle.” The RCMP were vehemently opposed to drug maintenance therapy. Abstinence and prison time were touted as the solution to addiction to narcotics. Halliday, an addiction specialist at the time, asserts that in Canada prior to the 1960s, the “absence of community treatment facilities must be directly related to the social concept of the addict as criminal first, and a sick person second” [33], p. 413. By the 1950s medical doctors gained new ground and psychiatric treatment in prison emerged as one response for new narcotic users [31,32,34].

Another perspective began to emerge in Canada in the 1950s, a drug treatment movement centred in Vancouver, B.C. Doctors, social workers, politicians, and concerned citizens rallied for change, including the setting up of drug maintenance programs for people addicted to narcotics [32,35]. Although they were not completely successful at that time, a number of small programs were later set up in Canada. These programs included limited methadone maintenance programs and some drug treatment programs in prisons, such as Matsqui prison in B.C. It was not until the 1960s and early 1970s that publicly funded methadone maintenance programs and drug treatment programs and other services became available throughout Canada, and even then rural areas did not have services and services in urban areas were quite restrictive [32,33].

Narcotic users have long questioned why heroin is illegal and why it is not offered as a choice for drug maintenance. From the 1960s on, methadone became the standard treatment in Canada for people addicted to narcotics and this treatment was expanded in B.C. in the late 1990s. Yet right from its inception, it was clear that methadone maintenance does not work for everyone. Research and later drug user groups made it clear that many narcotic users do not benefit from methadone therapy and many participants drop out or are kicked out of treatment for not complying with rigid regulations. Rather than methadone maintenance, people who participated in these programs requested heroin. But their requests fell on deaf ears.

However, heroin prescription is not unusual: the U.K. has long had heroin prescription as part of its addiction treatment services, and in the U.S., a number of heroin/morphine clinics were opened following prohibition. These public clinics were eventually closed down as the U.S. moved towards a more prohibitionist and criminal law model of drug policy and, at that time, little else was put into place to help people addicted to narcotics other than prison programs such as Lexington [36].

In the early 1950s, the *Senate Special Committee on the Traffic in Narcotic Drugs in Canada* visited Oakalla prison farm in Burnaby, B.C. This committee noted in its report that “without exception” all of the former narcotic users in the prison group advocated for the “legalized provision of drugs” [37], p. 344. The report also pointed out how medical and professional “addicts” were treated differently in Canada. From 1928 to the early 1970s, the Division of Narcotic Control (within the Federal Department of Health) kept a registry and case files on every known illegal drug user in Canada, whom they referred to as “criminal addicts.” Each file contained police reports, photos, criminal records, and memos and letters from doctors, prosecutors, and parole boards. Every arrest for narcotic possession or trafficking was noted in the files. The police also included notes from drug trials to inform the RCMP and special prosecutors about testimony and trial outcomes. Whenever the department found out that a known drug user was obtaining drugs from a doctor, they set out to investigate. A separate filing system was kept for medical professionals (doctors, nurses, pharmacists) known to use narcotics; yet to date, these files are unavailable and no researcher has been able to access them [31]. Most significant for the purposes of this paper, the two categories of narcotic user were treated differently: prison time was handed out to those labelled criminal addicts, and at the same time, medical professionals received private treatment and cautions.

There is a long history of advocating for people who use criminalized drugs, including legalizing narcotics in Canada and setting up alternative drug maintenance programs, such as heroin therapy. In the 1990s, Switzerland implemented heroin-assisted treatment (HAT) in several cities. The success of the Swiss program led to many other countries adopting similar models, including Germany, the Netherlands, Spain, Belgium, and Denmark. There is now a “rich data set on the feasibility, efficacy, safety and effectiveness of HAT” [38]. As noted above, heroin has not been available in Canada for maintenance purposes. In 1998, the first North American Opiate Medication Initiative (NAOMI) Working Group was formed to conduct a HAT trial in the U.S. and Canada. Because it was not possible to get a U.S. site for the trial, Canadian investigators applied for a 3-site study in Canada. Funding approval was granted by the Canadian Institutes of Health Research (CIHR) in 2002 followed by approval from the Regulatory Branch of Health Canada in 2003 [39]. NAOMI eventually opened its doors in only Vancouver and Montreal.

## NAOMI

In 2005 posters went up on telephone poles and walls throughout the DTES of Vancouver, with a telephone number to contact the NAOMI research people. NAOMI was a clinical trial that tested whether heroin-assisted therapy benefits people suffering from chronic opiate addictions who have not benefited from other treatments. The target population for NAOMI included men and women over the age of 25 who were “chronic, opioid dependent, daily IDUs” and who had previously been unsuccessful with methadone maintenance and other treatment modalities. Participants in the NAOMI study were randomized into one of two groups: one received injections of heroin or Dilaudid (hydromorphone), and the other received oral methadone. The NAOMI study provided heroin/Dilaudid for 12 months, followed by a 3-month transition period. When they entered the study, participants were not currently on methadone maintenance therapy (MMT) and had to be off MMT for at least six months prior to participating in NAOMI. Apparently this criteria for entering the study was added after response from Canadian health authorities and MMT providers who were worried that their patients would drop out in order to participate in NAOMI [39]. This is the opposite of participant criteria in other countries that actively recruited MMT patients. In fact the criteria of these other trials made clear the participants “must” currently be on MMT. The NAOMI trial began enrolling people in February 2005 in Vancouver, B.C. Recruitment ended in April 2007 and the last participants left the program in 2008.

## NAOMI study results

1. Heroin-assisted therapy proved to be a safe and highly effective treatment for people with chronic, “treatment-refractory” heroin addiction. Marked improvements were observed including decreased use of illicit “street” heroin, decreased criminal activity, decreased money spent on drugs, and improved physical and psychological health.

2. The NAOMI trial attracted the most chronic and marginalized heroin users who were outside the treatment system and continued to use heroin despite numerous previous treatment attempts. Both heroin-assisted therapy and optimized methadone maintenance treatment achieved high retention rates and remarkable response rates in this difficult-to-treat group.

3. Contrary to pre-existing concerns, the treatment clinics appeared to have no negative impacts on the surrounding neighbourhoods.

4. Participants on hydromorphone [Dilaudid] did not distinguish this drug from heroin. Moreover, hydromorphone appeared to be equally effective as heroin although the study was not designed to test this conclusively [40].

After a year of receiving heroin (or hydromorphone), participants entered a 3-month transition period. During this period, all NAOMI participants were offered a range of traditional treatments, including methadone maintenance and detox. After the 3-month transitional period, the research participants were no longer part of the study and no further treatment or supports were offered, although follow-up interviews were conducted 18 and 24 months following participants’ entrance into the study (thus, interviews occurred 3 months and 9 months after exiting the study). However, 16 percent of the total heroin/hydromorphone participants did not participate in the final follow-up interviews; thus it is difficult to assess the NAOMI researchers’ claims about participants’ medical status, treatment retention, drug use, legal situation, quality of life, etc. following cessation of treatment [41]. What is indisputable is that the NAOMI findings demonstrated that heroin-assisted therapy was an effective treatment that improved physical and psychological health when the participants were receiving treatment [39,41].

As early as 2006, Dan Small and Ernest Drucker questioned why both Canadian federal and provincial policy makers ignored the plethora of scientific evidence throughout the world that already demonstrated the efficacy of heroin prescription. They noted that the federal government of Canada rejected an early 2001 request by the Portland Hotel Society (PHS), a non-profit “social, health, and housing agency” in the Downtown Eastside, for legal permission to prescribe heroin in Vancouver, B.C. [42] Although they praise the NAOMI researchers for their efforts to provide heroin prescription in Canada, they also make clear that the NAOMI trial was problematic on many fronts, including the absence of an ethical exit strategy, the lack of informed consent without duress, and the failure to provide a permanent program [42], p. 11. They also point to the internationally accepted ethical standards for research outlined in the Declaration of Helsinki. The Declaration states: “At the conclusion of the study, every patient entered into the study should be assured of access to the best proven prophylactic, diagnostic and therapeutic methods identified by the study” [43]. An adequate exit strategy should have been included in the research proposal to transfer patients to a permanent program at the end of the NAOMI trial if the study results were positive (which they were). At the very least, HAT on compassionate grounds should have been provided for. Dan Small and Ernest Drucker assert that heroin prescription has improved the health and quality of life for participants around the world. They conclude that if the “research in another sector were as clear, this treatment protocol would by now be available” [42], p. 12.

The NPA participants also questioned why they were denied HAT and why such a successful trial would close down. Outside Canada, heroin-assisted therapy is offered in a number of countries and none of these programs shut down following their study stage; due to the fact that study results were positive, these programs continued on a permanent basis and/or participants were granted further HAT on compassionate grounds. For an excellent summary of HAT programs, see [44]. The Netherlands HAT randomized trial conducted over four years demonstrates that the longer a patient is offered HAT, the better is the chance of continued good health (in contrast to those patients with only one year of HAT) [38]. Continuation of HAT is thus essential. **The Canadian NAOMI project is the only heroin-assisted study that failed to continue offering HAT to its participants when the study ended in Vancouver**[44].^d^ In 2007, Health Canada refused compassionate use of heroin for NAOMI participants; however, Health Canada left the door open for the NAOMI researchers to continue providing heroin through their study. Yet, they failed to do so.

## NAOMI from the Perspective of the NPA

The preceding sections outlined some of the findings of the NAOMI trial from the perspectives of researchers involved in that study. This section of the paper is drawn from what the research participants said about their experience in the NPA focus groups about NAOMI. It highlights the experiences of the NPA members when they were NAOMI research subjects.

NPA members discussed why they chose to become NAOMI research subjects. Two members expressed their views:

*Well, we all wanted heroin. Everybody wanted the heroin*. (female NPA participant)

*That’s why we went through it all.* (male NPA participant)

Participants expressed why they were interested in participating in the NAOMI clinical trial. The trial offered something that participants wished to obtain: heroin, a drug currently criminalized in Canada.

NPA members also discussed the physical space of the NAOMI clinic over the study period. Participants in the study were expected to arrive at the clinic on the corner of Hastings and Abbott Streets three times a day (morning, afternoon, evening) with about four hours between each dose. The people who received heroin came in the entrance on Abbott St., and the people receiving methadone entered on Hastings St. The two groups remained physically separate during the study. When the heroin participants arrived, they had a 10-minute window for their appointment. They were not allowed to arrive early or to be late.

*They didn’t want people lining up*. (male NPA participant)

*Okay? Not 10 minutes and 10 seconds. You had 10 minutes . . . . The computer would not allow you to be logged in if you were past that 10-minute window*. (female NPA participant)

Participants were buzzed though a double door that had security cameras; the doors were continuously locked. People then had to be buzzed through a second door. Patients were then logged on to a computer and they entered a waiting room for a 15-minute observation prior to injecting their dose of heroin or Dilaudid. After being observed for 15 minutes, on a first-come-first-served basis, participants were brought into the injection area (similar to Insite) where a nurse sat behind a glass partition and supplies were handed to people in a tray, including a prepared syringe that was scanned to match each participant’s name. Then they had seven minutes to inject, followed by a half-hour observation period following their dose (3 × day). Participants sat in a lounge during this observation period.

*You were given seven minutes to inject your heroin*. (male NPA participant)

There were nurses and social workers observing people, and a doctor was on site. Every two weeks the participants met with the doctor to discuss their dose. They could also arrange for a meeting in between these appointment times.

The NAOMI participants spent a lot of time at the clinic waiting for their medication and being observed before and after their dose. In that time, they talked with one another, formed friendships, and created activities to fill in the time. One NPA member wrote about her time waiting at NAOMI with other participants:

Supper at NAOMI

It’s 5pm, time to go to my regular evening medication of heroin. All done with the medical part. Now to the fun stuff. Dave brought a Maple Leaf roast pork and gravy dish, fed about 8 people, the 4 at our table and then as many as we can. I brought bread and salad. So dinner tonight is:

“Hot Pork Sandwich – with gravy, Caesar Salad and Vanilla pudding with Strawberries”

Supper’s over, I got fed and now it’s time to relax and do our crosswords.

*Bye! See you for breakfast.* (Dianne, NPA member)

NPA members noted that by abiding by the protocols and regulations set up by NAOMI for their attendance, they spent a lot of their day at the clinic and it was difficult to do anything outside the clinic. One NPA member said:

*Well, you couldn’t do anything in between.* (male NPA participant)

## NAOMI benefits

The NPA members spoke about the benefits they experienced during their time as participants in the NAOMI trial:

*They helped me get a room at the Empress Hotel and from there everything started to move forward. I didn’t have to worry about having to get up every morning and run all over hell’s half acre just like a chicken with my head cut off wondering where I was going to get the money to get better.* (female NPA participant)

*And life improved, I suppose. It was kind of a blurry year but all in all I think it was better. I don’t know. I was happy, at least I think I was happy. You know, I wasn’t miserable a lot. I wasn’t sick, you know, I wasn’t running around trying to get $10 all the time. Yeah, so I mean it was good.* (male NPA participant)

*I am glad I did go through it even if we did get dropped because it was, it was the best years of my – couple of years in my life. I really learned how to be myself without having to be looking for money all the time. I learned how to do normal things, and be a good president [at VANDU] and stuff.* (female NPA participant)

I was in a bubble for 15 months, but I mean, I hit those doors at 72 pounds and, you know, here I am now and it’s not – NAOMI. I had a hell of a good time. I was helped with housing. When I got an abscess on my hip the nurses from NAOMI were pulling up at Powell Place and coming and getting me and driving me without me even asking them.

But it changed my life a lot. I wouldn’t regret doing it. I’d do it again if they would offer it again. It was a good thing for me while it lasted. It was great. It was just good for me because I just can’t do methadone. It is not an option for me. (male NPA participant)

It would give me a huge break in my life as an addict. It would give me this huge, like, vacation, that was like going to Florida, you know, and living on the beach almost, you know, in terms of addiction. Going to Florida. (male NPA participant)

The NPA members reveal that the benefits of the NAOMI trial were deeply felt, as were the effects of not having to hustle every day. However, some NPA members were not able to comply with the requirements of the NAOMI trial:

*I was only on it for three months, but during the three months my life got a lot better and when I did get kicked off it my life was kind of screwed up because I’d forgotten how to hustle to get, you know, to get things happening.* (male NPA participant)

Others observed that they were worried about what would happen once the clinical trial ended:

*We were being observed, the 15 minutes and a half hour after, so there was a lot of talking going on and I think one of the big subjects was, what are you going to do when this was over? And I think that was, like, probably the thing we talked about the most.* (male NPA participant)

*I mean, once you finished talking about your daily activities or whatever was going on in your life it came down to, like especially as it got closer to the end road. I think that it was helped by the fact that it was staggered, the intake, so we would always have somebody that was leaving…so we had an idea it was coming all the time…*(male NPA participant)

NPA members expressed their concerns about the end of the NAOMI clinical trial. One woman noted:

I just – you know, I cried like a baby the last day I was there. (female NPA participant)

The lack of an adequate exit strategy for the patients was sorely felt and is discussed more fully in the next section.

## Consent and expectations: what is consent under drug prohibition?

All of the NPA participants signed consent forms to participate in the NAOMI study. These forms were updated from time to time. The NPA members discussed issues of consent, ethics, and their expectations of the NAOMI trial. They also felt optimistic that the study would eventually become a permanent program.

*I went there with the full understanding it was a study. It was a study.* (female NPA participant)

*The staff and the doctors were telling us no [the study would not continue], but they never completely extinguished that little dream that we had. . . . We were optimistic.* (male NPA participant)

*I was given the impression that it would continue and then the studies that had happened in Europe that all –they’d all been on compassionate ground, so I really thought it was going [to continue] …It really kind of threw me for a loop when it didn’t happen that way.* (male NPA participant)

I knew it was a study and like everybody else it was going to help the future generation. But for me it was a double-edged sword . . . . I think the thing that’s flawed in this, in the ethics, was the ethics approval, like, for them to approve this study without fully – I mean, without having an exit strategy in place that was doable.

*There’s been these kind of studies done in other countries before us. So they had a good idea of what the results were going to be . . . And to go into that without having a way out that worked for the client or the participant, I think that was the -- that’s the thing that wasn’t right, in my opinion.* (female NPA participant)

Several of the NPA members explained how consent is problematic when researchers control the very drug that is an integral part of their lives:

*Our life depends on this drug and here we’re offered this drug. Well, okay, so, I mean, I always said, well, I would sign anything at that point.* (male NPA participant)

The NPA members also wondered why the positive NAOMI trial findings were not fully considered by the federal and provincial governments. They wondered if the failure to create a permanent program had to do with their marginalized status as illegal drug users. The NPA members noted that if a diabetes or cancer treatment proved to be efficacious during a clinical trial, presumably the patients would continue to receive the medicine or treatment.

*If they give you a drug for – they’re experimenting with a drug for cancer and it starts working. I mean, what are they – what are you going to do? Oh, no. You can’t have it anymore, we’re going to back off here.* (male NPA participant)

In contrast, the NAOMI patients were denied heroin-assisted treatment when the trial ended.

The NPA members also noted that providing legal access to heroin or any drug improves the lives of the user. However, that in itself is not enough:

*If you just give me the drug all the time are you improving my life? Well, you’re improving my life as far as the drug goes. You’re probably taking a lot of the stress out of my life, but are you actually doing these other steps?* (male NPA participant)

The NPA members discussed how Canadian studies rarely provide necessary social and economic supports or lead to social change. Nor does drug policy necessarily change as a result of these studies. However as the following NPA participant notes, providing HAT along with social and economic supports opens up more positive possibilities:

*And then we could move forward in our lives.* (female NPA participant)

## NPA recommendations for future “experimental” drug maintenance programs

Drawing from the experiences of NPA members at weekly meetings, the brainstorming session, and focus groups, the NPA also developed recommendations for future experimental drug maintenance studies and programs. They are as follows:

When experimental drug maintenance programs are over, clients (research subjects), for compassionate reasons, should receive the drug they were on as long as they need it.

An ideal study would provide an umbrella of support and services:

● Housing (most important)

● Access to medical treatment all under one roof (nurses, family doctors, dentists, etc.)

● Access to welfare workers (who are familiar with the area and the people who live there) and Ministry representatives

● Access to nutritious food for self and family

● Support to move life forward (school, trade, family unification)

● Access to lawyers

● Education/advocacy skills and access to advocates

● Diverse routes of administration available—oral, smoking form, injection. Not all people want to inject their drug.

An ideal study would utilize the time clients (research subjects) spent on site, three times a day. This considerable amount of time could be used to support, educate, and advocate. All future studies and programs should include an adequate exit strategy, and NPA and other heroin users should be part of the team from the beginning.

## Following NAOMI: snapshot of the NPA, where they were in 2011

Dave Murray, the founder of NPA, gathered the following information on housing on June 11, 2011. Out of the 13 people present at the NPA meeting that day:

● seven lived in SROs (single room occupancy)

● two lived in social housing (specifically Woodward’s social housing)

● four self identified as homeless (no fixed address)

Most of NAOMI participants (IDU side) were housed in SROs during the study period. No official effort was made by the study to assist them to get better housing during or after the research project. Everyone present at the Saturday NPA meeting said that they would have liked help (B.C. Housing, etc.) to improve their living conditions during the study (and today). The two people living in Woodward’s (social housing) today were in SROs during the study.

NPA member and friend Robert Vincent passed away in late December 2011. His thoughts and writing are included in the 2012 Report. In the Report, the NPA also honoured the memory of other former NAOMI participants who have passed away [1]. The NPA continue to meet every Saturday at VANDU and they continue to offer support and advocacy to their members. They are also active in trying to change drug policy, especially in relation to HAT.

## HAT in Vancouver

In December 2011, the SALOME (Study to Assess Longer-term Opiate Medication Effectiveness) study opened its doors in Vancouver. SALOME is another clinical trial. Their website describes the study as testing whether hydromorphone (Dilaudid), “a licensed medication, is as good as diacetylmorphine, the active ingredient of heroin, at benefiting people suffering from chronic opioid addiction who are not benefiting sufficiently from other treatments. This study will also test if those effectively treated with these two injectable medications can be successfully switched and retained to the oral formulations of the medications” [45].

In order to test their hypothesis, the SALOME study will compare the effectiveness of six months of injectable heroin with six months of injectable Dilaudid and the effects of switching from injectable to oral heroin or Dilaudid. However, NPA members question why the SALOME trial will compare Dilaudid to heroin given that HAT is already proven to be feasible, safe, and effective [38]. SALOME began active recruitment in Vancouver, B.C. in December 2011. Utilizing a lottery system, people who registered for the trial and were deemed eligible were contacted. Participants will be in the study for one year, followed by a 1-month transition period where participants will be encouraged to participate, once again, in conventional treatments such as methadone maintenance, drug-free treatments, and detox programs (treatments that have proven to be ineffective for these participants). The repeated failure of treatment efforts for participants is in fact part of the criteria for selection of participants in SALOME, as was the case in NAOMI.

In 2011 and 2012 the NPA met with SALOME researchers prior to their recruitment of research subjects and provided valuable input from their experiences as NAOMI patients. They also shared with SALOME researchers their recommendations for future HAT trials and programs as outlined above. NPA findings were also communicated at public events at Simon Fraser University Woodward’s in November 2011 and VANDU in February 2012, in Vancouver, B.C. However, in the end, the SALOME researchers failed to put into place the most significant recommendations of the NPA outlined in this paper. In the pursuit of scientific evidence, important issues and recommendations by the NPA about consent, ethics, an adequate exit strategy, and human rights have mostly been ignored. It must be noted that the SALOME study also has no exit strategy in place for its participants [45]. Thus, history may repeat itself in Canada.

NPA members assert that both the NAOMI and SALOME clinical trials should have included an exit strategy contingent on the efficacy of HAT, especially given that international research on HAT supported such an outcome. In other words, both studies should have offered a provision for continuing treatment (if they showed beneficence) for the participants. Continued treatment should have been built into the research protocols. Instead, at the end of the 12-month period, NAOMI participants had 3 months (and SALOME provides one month) to transition to other available conventional treatments, such as abstinence based programs and methadone maintenance treatment. Yet, as noted earlier, failure to benefit from traditional treatments was one of the criteria for participation in NAOMI, and now SALOME. The NPA argues that adequate exit strategies should have been built into the clinical trial and that withholding treatment that proved effective for patients is unethical. Furthermore the NPA members question the responsibility healthcare investigators have to their subjects in these studies; are they not “patients” rather than merely “subjects”?

In response to questions raised by NPA about the failure to provide an adequate exit strategy and provisions for a permanent HAT program, both NAOMI and SALOME researchers have recently pointed to the complexities of the application process for HAT clinical trials in Canada, communications and restrictions imposed by Health Canada, site-specific security concerns, and funding. The researchers also note that they did receive ethics approval for their trials to proceed [41]. Yet, none of their explanations address the lack of an adequate exit plan, nor do they fully explain their failure to continue HAT for their patients. Nor do their explanations address the ethical issues discussed by NPA members in this article.

In this paper, NPA members express the benefits and problematics they experienced during their time as participants in the groundbreaking NAOMI trial. Also, consent is contextualized. Consent is not only about ability or mental competency to provide consent. The NPA members made clear in the focus groups that they gave consent to participate in the NAOMI study. However, the NPA points to other ethical issues related to consent that are not addressed by the NAOMI researchers. The NPA illuminates how consent is compromised when participants are vulnerable and marginalized, and essential social and economic supports are lacking for patients. They ask readers to consider some of the social conditions that shape their live in the DTES: economic marginalization, poverty, discrimination, violence, police profiling, lack of housing, drug prohibition and the criminalization of heroin, and inflated black market prices for their drug of choice. As one NPA member stated earlier: *Our life depends on this drug and here we’re offered this drug. . . . I always said, well, I would sign anything at that point.* He continues: *I would probably say which finger do you want, you know, or which arm do you want, you know.*

Furthermore, the NPA members noted that if a cancer drug proved to be efficacious during a clinical trial, would the patients be denied the drug at the end of the trial? The NPA members question why it is ethical to deny NAOMI and SALOME patients the best medicine — HAT. The NPA contends that the NAOMI researchers should have pushed policy makers harder to allow for on-going clinical prescription. They also assert that SALOME researchers and Providence Health Care should do the same now. As noted earlier in this paper, Canada is the only country that did not continue to provide HAT to its patients following NAOMI.

Hoping to avert the same outcome with the SALOME trial and any other proposed HAT trials, the NPA with the British Columbia Association of People on Methadone (BCAPOM) consulted with Pivot Legal Society, a non-profit located in the DTES of Vancouver, B.C., whose “mandate is to use the law to address the root causes of poverty and social exclusion” [46]. They are working closely with Pivot to create a different outcome, to assure that the SALOME trial immediately becomes a permanent heroin treatment *program*. Pivot asserts that should the federal government erect a barrier to the recommended shift to a heroin prescription program, the recent decision of the Supreme Court of Canada in the Insite (Vancouver’s safe injection site) case “supports the argument that the federal government cannot deny a legal exemption to the Controlled Drugs and Substances Act for patient health care outside of a research setting when evidence demonstrates the efficacy of such treatment. To do otherwise would breach the Charter rights of patients needing medical care” [47].

## Conclusion

The NPA started a small revolution in Vancouver, B.C. It is too soon to know the full outcome of their advocacy; however, the NPA hopes that this paper and their earlier Report [1] will guide future research studies and the setting up of permanent heroin maintenance programs in Canada and elsewhere. This paper provides insights into the lives of the people who became research subjects when they participated in the NAOMI clinical trial. The NPA members advocate for the end of drug prohibition so that other people will not be subject to the social and legal discrimination that they face daily. Nor will people feel compelled to participate in research projects in order to have essential goods, drugs, services, and supports provided to them. The NPA encourages other groups to engage in creating their own research to tell their own stories to improve the lives of those most affected by drug prohibition and to guide future programs that offer supports and substitution drugs to users.

## Endnotes

^a^ In the fall of 2012 the NPA informally changed its name to the SALOME/ NAOMI Association of Patients (SNAP), to better reflect their current membership. A number of NPA members are currently participating in SALOME, a clinical trial in the DTES. However, for the purposes of this paper, the authors refer to NPA because it was in use during the initial NPA research period.

^b^ The writing workshop took place on June 11, 2011 and 14 NPA members attended. Thirteen people contributed writing pieces (5 women and 8 men).

^c^ Opium is made from the opium poppy. It is one of the oldest drugs recorded, the parent of all other narcotics. It was an important item of commerce and used widely for medical purposes. 

^d^In addition, the SALOME website states that: “Canada will be the only country that has ever terminated the treatment after showing success. The Canadian study team applies for research funding to continue investigating effectiveness of licensed injectable opioids (the SALOME trial).” Timeline: From Opium to Salome. [http://www.providencehealthcare.org/salome/timeline.html]

## Competing interests

The authors declare that they have no competing interests.

## Authors’ contributions

Both Susan Boyd and the NAOMI Patients Association collaborated in the research process and drafted the manuscript. Both authors (SB and NPA) read and approved the final manuscript.
